# Retrospective analysis of custom 3D-printed drill guides and titanium plate use in spinal stabilization of eleven dogs

**DOI:** 10.3389/fvets.2024.1491620

**Published:** 2024-12-20

**Authors:** Phillip Guirguis, Ilja Asanovic, David S. Beggs, Sam N. Long

**Affiliations:** ^1^Melbourne Veterinary School, Werribee, VIC, Australia; ^2^Centre of Advanced Batch Manufacture, Swansea, United Kingdom; ^3^Veterinary Referral Hospital, Dandenong, VIC, Australia

**Keywords:** canine, vertebral malformation, spinal stabilization, titanium plates, 3D printed drill guides

## Abstract

**Introduction:**

Congenital vertebral malformations are common developmental abnormalities in screw-tailed brachycephalic dog breeds. Subsequent vertebral instability and/or vertebral canal stenosis caused by these malformations can lead to spinal cord compression manifesting in pain, paraparesis, ataxia and/or paralysis. Various methods for spinal stabilization are in common use. However, these are without significant risk due to narrow margins of surgical error and variable vertebral anatomy. We evaluate a novel method for spinal stabilization where a custom 3D-printed plate is created and surgically fitted to the patient’s spine using custom 3D-printed drill guides.

**Objective:**

To describe the surgical technique and short-term outcomes in patients treated with custom 3D-printed plates and drill guides.

**Method:**

A retrospective analysis of 11 dogs from two referral hospitals which underwent this procedure was undertaken. Post-operative CT scans were assessed for spinal canal screw perforation using the modified Zdichavsky classification. Pre-operative and post-operative neurological status were assessed using the Modified Frankel Scale and the surgical technique including post-operative imaging and recovery findings were described.

**Results:**

Optimal screw placement (grade I) was achieved in 63% of placed screws across the eleven dogs. Partial penetration of the medial wall (grade IIa) was observed in 3% of screws and partial penetration of the lateral wall (grade IIIa) was observed in 29% of screws. Full penetration of the lateral pedicle wall (grade IIIb) was observed in 5% of screws and no screws fully penetrated the medial vertebral wall (grade IIb).

**Discussion:**

We demonstrated that custom 3D-printed drill guides and titanium plates can provide a safe peri-operative alternative for surgical spinal stabilization of dogs with vertebral column instability due to congenital vertebral malformations. Further research is needed to describe long-term outcomes of this surgical technique on patient health.

## 1 Introduction

Congenital vertebral malformations are developmental vertebral abnormalities commonly reported in screw-tailed brachycephalic breeds such as the English Bulldog, French Bulldog and the Pug (1–4). Frequently observed malformations include hemivertebrae (lateral, ventral or ventrolateral aplasia), wedge vertebrae (lateral or ventral hypoplasia) and butterfly vertebrae (ventral and median aplasia) (4, 5) although not all types of malformation fit into these categories (6). Together these malformations result in large scale abnormalities such as kyphosis, lordosis, scoliosis, or a combination of these. In addition, malformations affecting the dorsal part of the vertebrae have been reported, resulting in hypoplastic or aplastic articular facet processes, occurring more commonly in Pugs (7). Previous studies have reported the incidence of congenital vertebral malformations in Pugs to be as high as 96% and hemivertebrae prevalence in French Bulldogs to be up to 94% (8). The incidence of articular facet dysplasia has been reported to be 71% in French bulldogs, 84% in English bulldogs and 97% in Pugs (7). Whilst a definitive cause and pathogenesis for congenital vertebral malformations is unknown, previous studies have proposed a multifactorial aetiology including genetic factors, teratogen exposure impacting cartilaginous proliferation and a congenital absence of vertebral vascularization (3, 4, 6). A proposed genetic defect involves a variant of the DISHEVELLED 2 gene. This gene is responsible for screw-tailed anatomy and has been found to segregate with thoracic vertebral malformations, providing a possible explanation for increased vertebral malformation frequency in screw-tailed breeds (9).

Many cases are subclinical, but they can also result in neurological deficits such as paraparesis, pain, ataxia and paralysis, as the result of vertebral canal stenosis and/or vertebral instability (10–13). Spinal hyperesthesia occurs much less commonly (14). Although these vertebral malformations are present at birth, they typically do not become clinically apparent until 4 to 10 months of age when periods of accelerated growth result in increased spinal curvature (11, 15, 16). Canal stenosis tends to occur secondarily to malalignment and angulation of the spine when kyphosis, lordosis, and scoliosis are present, potentially in association with additional compression from an intervertebral disc, and is more likely in cases with a greater Cobb angle (4, 5, 8, 17). Several studies have suggested that there is an increased likelihood of neurological signs in pugs with both hemivertebra and a Cobb angle exceeding 35 degrees (3, 4, 8, 18, 19). In contrast, patients with articular facet abnormalities tend to present with additional spinal cord lesions such as intra-arachnoid diverticula, constrictive myelopathy, or with T2-weighted hyperintensity within the spinal cord presumed to be either gliosis or oedema (20, 21).

Currently, most surgical treatments described for treating congenital vertebral malformations involve spinal fixation with or without decompression (14, 22). Common methods for spinal fixation include pins (monocortical or bicortical) and polymethylmethacrylate (PMMA), spinal stapling, vertebral body plates (String of Pearls or Locking Compression Plates) and external skeletal fixators (ESF) (23–28). With the exception of spinal stapling, almost all described techniques involve the placement of either screws or pins into the vertebral bodies or pedicles. Whilst these surgical procedures can provide anatomic stabilization and rigid fixation to the affected vertebrae, there is a significant iatrogenic injury risk to neurovascular structures, nerve roots and the spinal cord. This has been quantified in several studies in which the rate of spinal canal violation has been described, ranging from 92 to 100% with bicortical pin use despite appropriate recognition of anatomical insertion and angle landmarks (29–31). A separate study found that only 48% of implants placed with a freehand drilling technique were considered to have acceptable placement compared to 64% for screws placed with the aid of 3D-printed drill guides (32). This is because freehand drilling is largely blind beyond the surface of vertebrae and the surgeon is only guided by approximate qualitative visual and tactile feedback when drilling (33). Additionally, existing literature states that risks of perforation of the spinal canal or implant failure such as screw pull-out are considerable in veterinary medicine (34, 35). Neuronavigation systems have been developed to assist in implant placement by allowing the surgeon to visualize real-time screw placement and have shown promise in implant placement accuracy and reduction in iatrogenic injury risk, however, these navigation systems have been reported to have a steep learning curve and high associated cost (33). Other improvements such as 3D-printed patient-specific drill guides (3D-PSDG) have been used to allow safe screw trajectory into the vertebral bodies as they ensure placement of drilling holes are in accordance with pre-operative calculated measurements and trajectories. One recent study found that 169/201 screws were adequately placed with the assistance of 3D-PSDG whilst another study found that 64% of screws placed with 3D-PSDG had acceptable placement, however, structures such as rods or PMMA to join the screws are still needed (32, 36). For this reason, there are potential opportunities for safer and more secure methods for achieving spinal stabilization.

In recent years, technological advancements in 3D-printing have led to the ability to print spinal stabilization plates that can be composed of implantable polymers or titanium (22). In this study we describe a novel spinal fixation system that comprises 3D printed titanium plates designed specifically for individual patients, in conjunction with a 3D-PSDG which ensures accurate and safe placement of the screws required to secure the plate in place. We describe a series of eleven dogs with a variety of vertebral malformations in which these plates were applied and detail the surgical technique, the rate of spinal canal violation, postoperative imaging findings and postoperative recovery.

## 2 Materials and methods

### 2.1 Patient details

**Figure 1:**
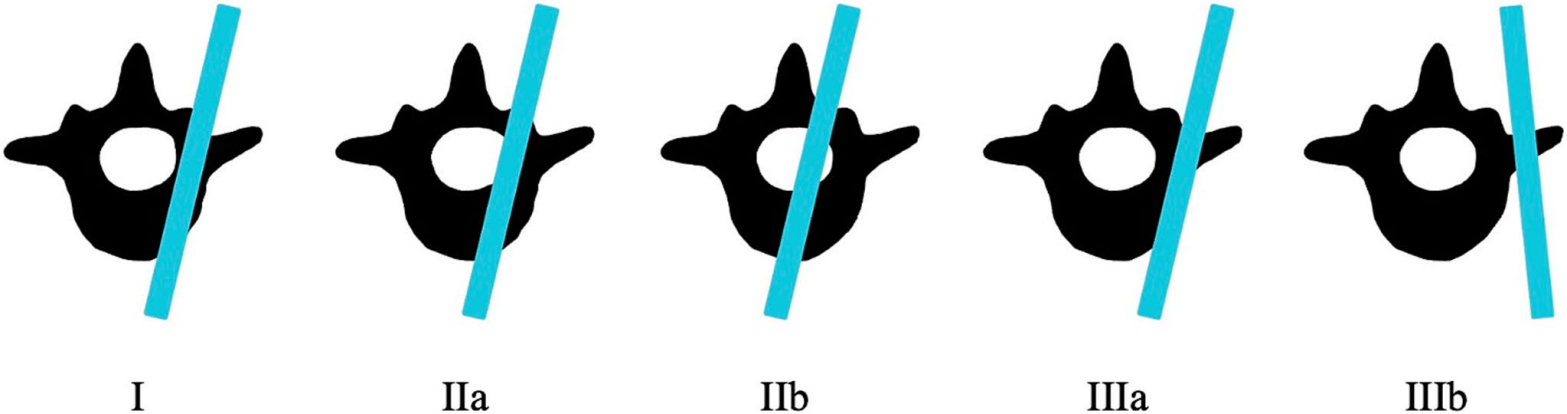
Screw placement using the modified Zdichavsky classification.

Medical records of eleven dogs from two small animal referral hospitals who were treated surgically with 3D-printed spinal plates and drill guides specifically designed for their individual malformations (CBM Medical, Swansea, UK, https://www.cbmwales.co.uk/our-services/medical-services/) were reviewed retrospectively. Patient data on breed, age at presentation, sex, neuter status, body weight and pre- and post-operative neurological status was included in this study. Pre- and post-operative neurological signs were graded using a Modified Frankel Score (MFS) system that has been previously described (Table 1) (36). Information regarding patient faecal and urinary incontinence was collected. Post-operative CT scans were examined, and the number of spinal canal screw violations were counted for each patient. Screw placement was graded based on a modified Zdichavsky classification described previously (Table 2 and Figure 1) (36). All patients were treated by the same ECVN board-certified neurosurgeon (Author, SL).

**Table 1:** Modified Frankel Score (MFS) for neurological signs.

Grade	Neurological signs
1	Spinal hyperesthesia only
2	Ambulatory paraparesis
3	Non-ambulatory paraparesis
4	Paraplegia with intact pain perception in both pelvic limbs and tail.
5	Paraplegia with absent pain perception in both pelvic limbs and tail.

**Table 2:** Modified Zdichavsky classification for screw placement.

Classification	Description
I	Optimally placed pedicle screw fully contained within the pedicle and vertebral body.
IIa	Partial penetration of the medial pedicle wall.
IIb	Full penetration of the medial pedicle wall (whole of screw diameter within vertebral canal).
IIIa	Partial penetration of the lateral pedicle wall.
IIIb	Full penetration of the lateral pedicle wall (whole of screw diameter outside the vertebral canal).

As this was a retrospective analysis of dogs treated in the normal course of practice, animal ethics approval was not required.

### 2.2 Imaging

All patients had both MRI and CT scan performed in order to diagnose their condition and determine the site of stabilization. MRI scan was performed on either a 3 T MR-PET or 1.5 T magnet (Siemens Biograph MMR, Siemens Medical Solutions, Erlangen, Germany, and SIGNA Creator; GE Healthcare, Milwaukee, Wisconsin, USA). MRI sequences varied slightly but all patients had the following sequences performed as a minimum: Sagittal and transverse T2W, sagittal and transverse T1W, sagittal and transverse T1W with gadolinium intravenous contrast (0.5 mmol/mL; sGE Healthcare, Milwaukee, Wisconsin, USA), and dorsal STIR sequence. CT scan was performed on a 16-slice CT (GE Lightspeed, GE Healthcare, Milwaukee, Wisconsin, USA and Siemens Somatom Sensation, Siemens Medical Solutions, Erlangen, Germany) with both bone and soft tissue window processing.

### 2.3 Drill guide and spinal plate creation

CT scan of the patient’s affected vertebrae was performed and using Digital Imaging and Communications in Medicine (DICOM) software, a triangulated mesh model of the vertebrae was obtained. The triangulated mesh model was then exported to CAD software for determination of optimum screw size, placement, and orientation. Digitally represented cylinders of equal diameter to the screw were used to plan the ideal screw trajectory into the vertebrae. A drill guide was then designed using an inverted virtual representation of the vertebrae. The screw trajectories were then mapped into the drill guide and guidance sleeves were created around each screw trajectory. The designed drill guide was then exported and printed via 3D-printing software. The material used for the 3D printed drill guides was methacrylate photopolymer resin. The drill guide was then autoclaved and used intraoperatively to assist in pedicle screw placement.

**Figure 2:**
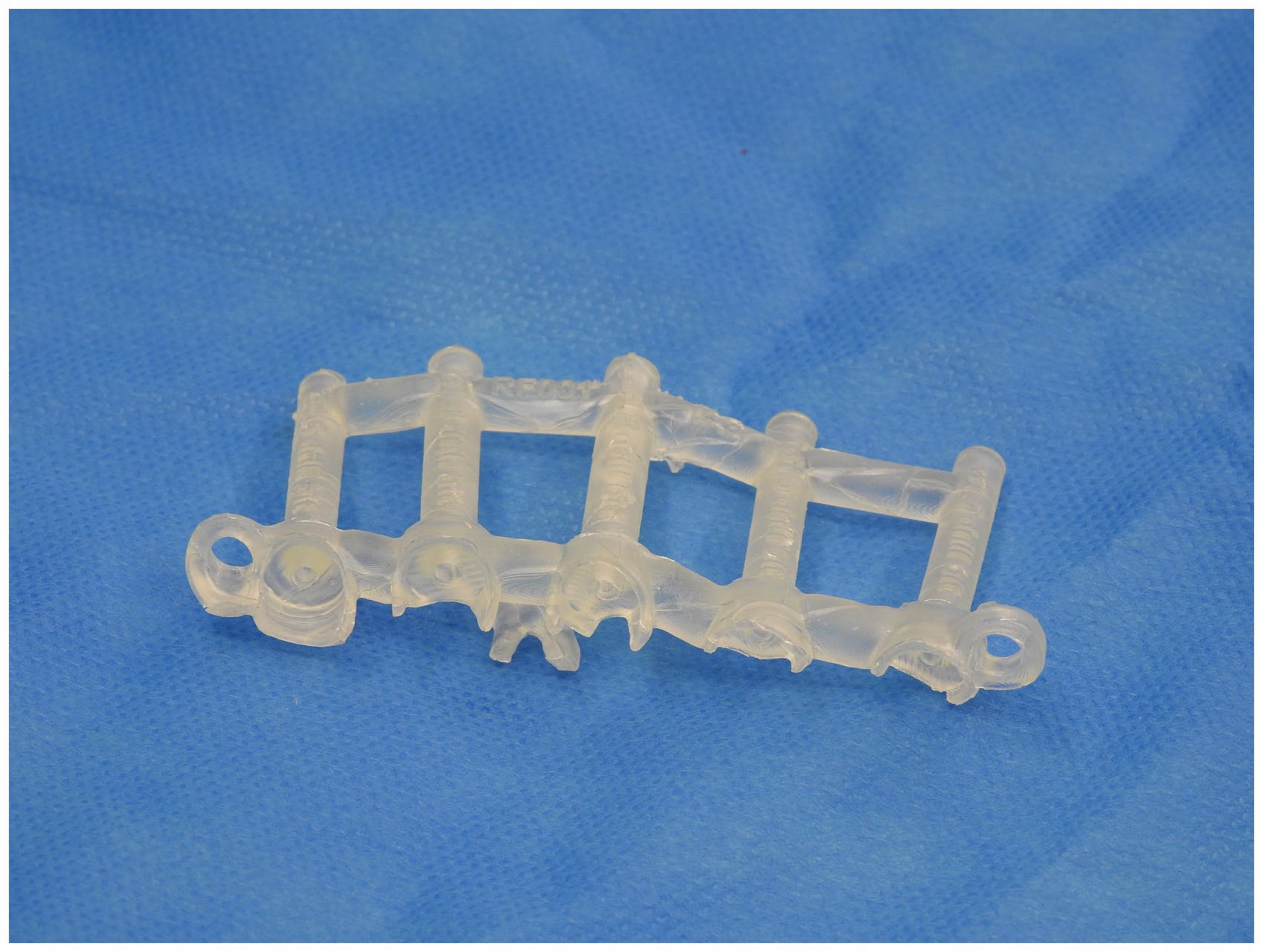
3D-printed drill guide.

**Figure 3:**
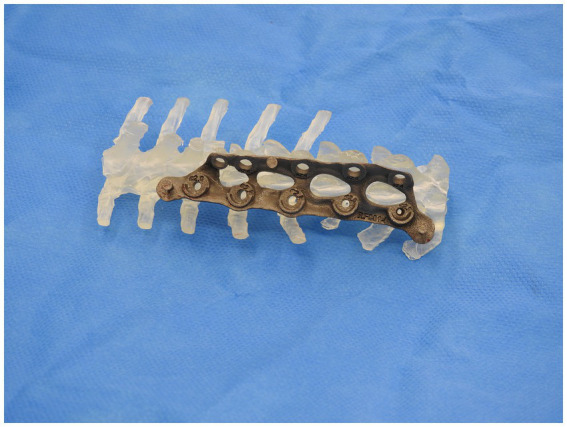
3D-printed spinal plate attached to a model of patient’s spine.

**Figure 4:**
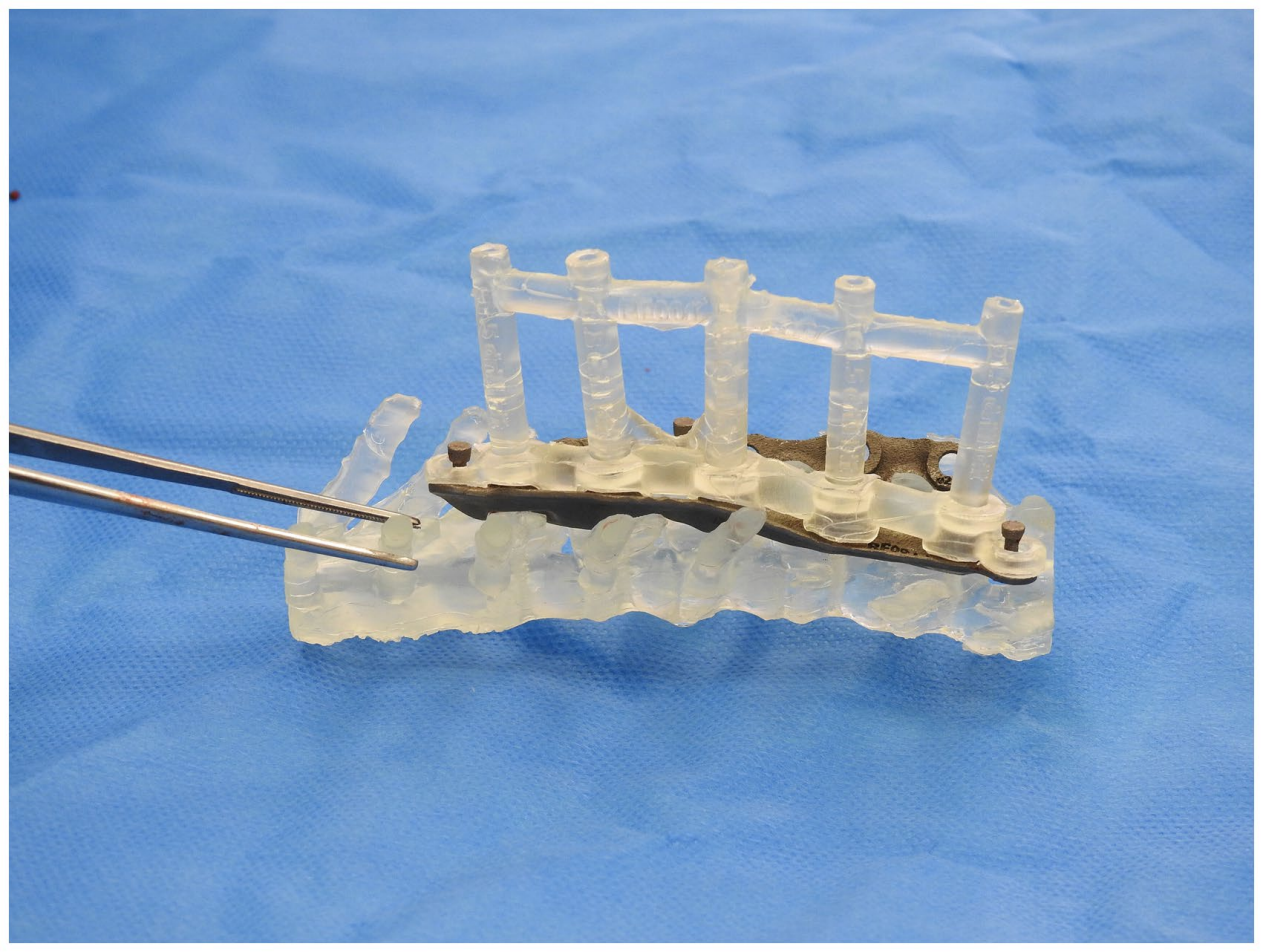
Drill guide and spinal plate attached to a model of a patient’s spine.

A similar process was adopted with the creation of the spinal plate. This involved using the DICOM images of the CT scan of the patient’s spine to create a template of the vertebral column. The template was then exported to CAD to allow for the design of the patient-specific implant. The implant design was then exported to a 3D-printing software and was printed on titanium. Figures 2, 3 depict the drill guide and spinal plate, respectively. Figure 4 depicts the drill guide and spinal plate attached to a model spine.

### 2.4 Surgical procedure

All dogs were anaesthetized using a standard protocol which included premedication with an opioid and co-induction with midazolam (0.2 mg/kg) and either Propofol or alfaxalone (4 mg/kg or 1.4 mg/kg respectively) and maintained with isoflurane and oxygen. A standard approach for a dorsal laminectomy was made to the affected region and a mixture of blunt and sharp dissection conducted to expose the spinous processes, rib heads, laminae and articular facet joints of the vertebrae of concern. Careful attention was paid to the removal of as much soft tissue as possible from bony structures to allow sufficient contact with the plate.

**Figure 5:**
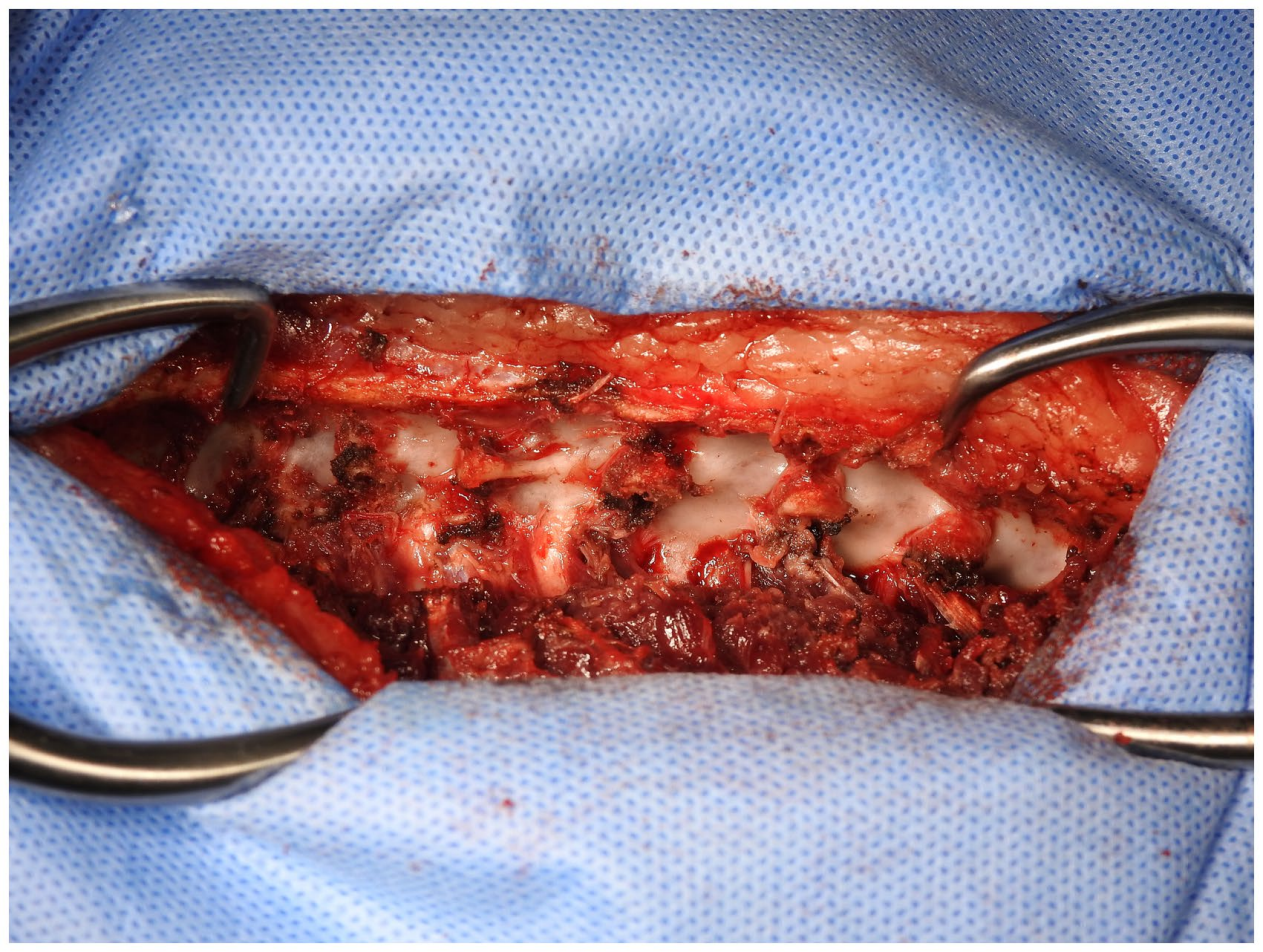
Exposure of vertebral bodies following removal of soft tissue.

The custom 3D-printed plate was fitted in three stages. In the first stage, following exposure of the bony structures of the patient (Figure 5), the plate applied to the vertebral bodies and spinous processes and checked for fit to ensure no movement of the plate was possible when in position. If necessary, further soft tissue dissection was performed to ensure good contact and registration between the patient’s bony anatomy and the plate.

**Figure 6:**
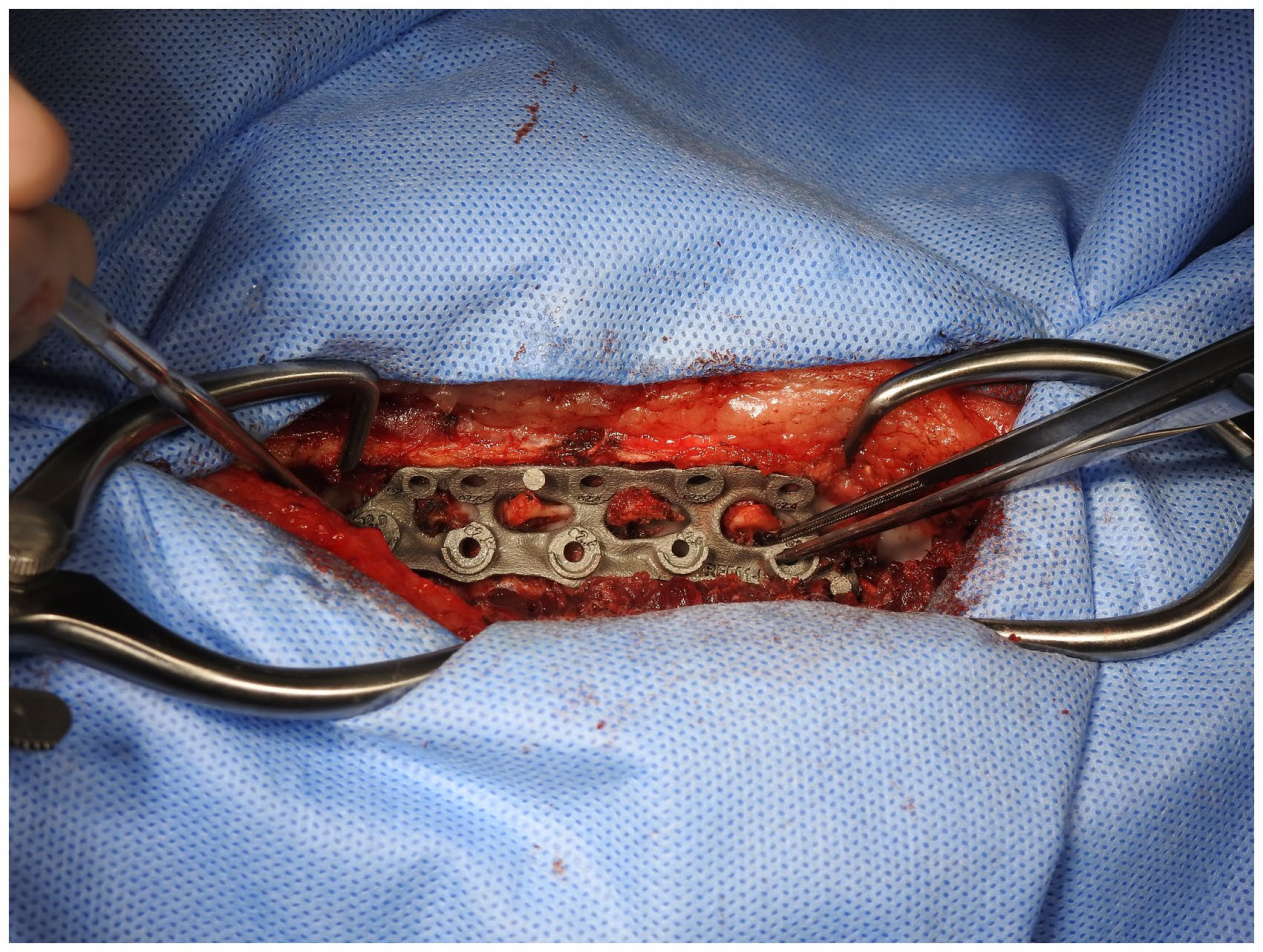
3D-printed Titanium plate held to the patient’s spine with haemostats.

**Figure 7:**
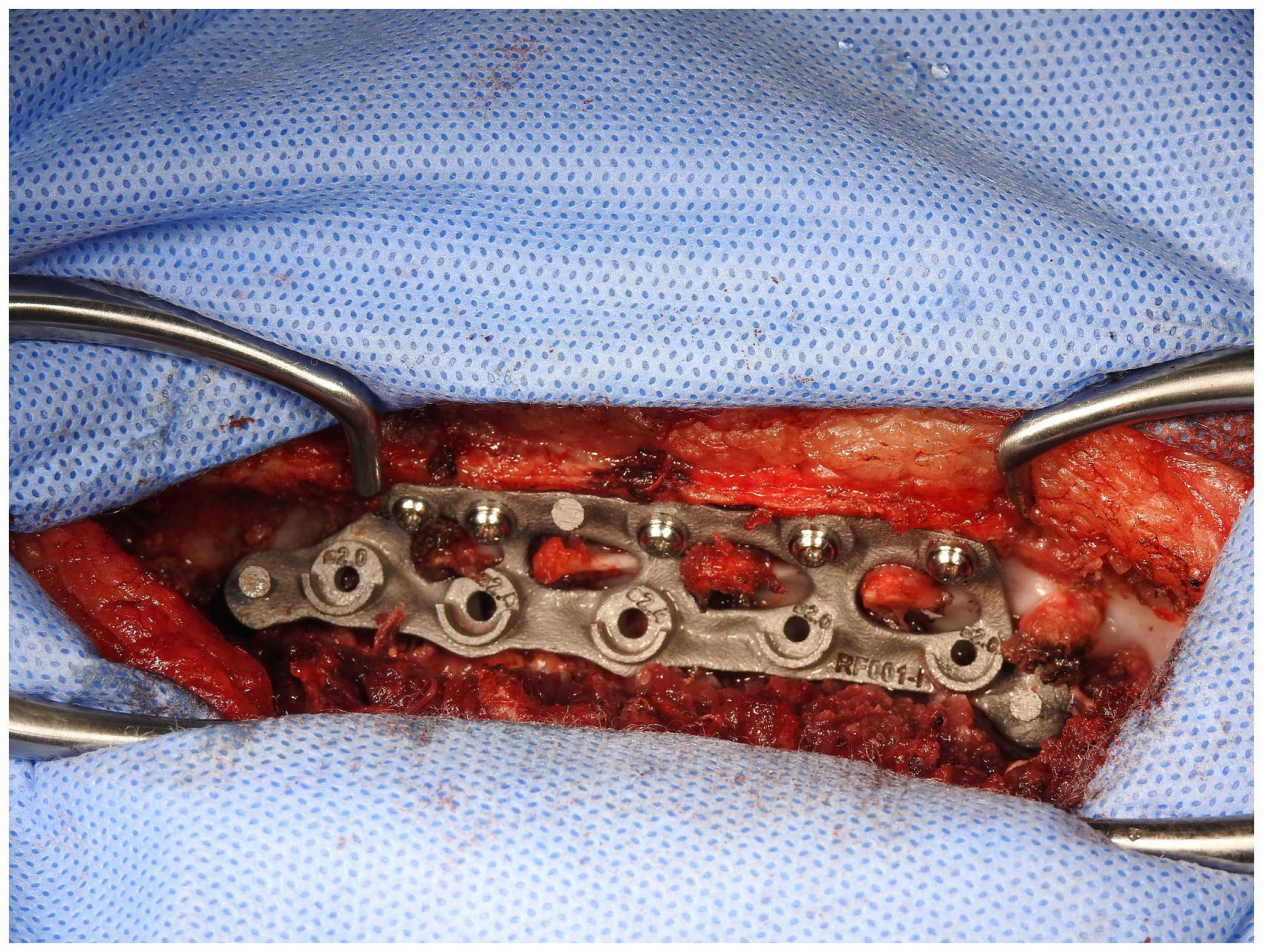
Screws placed to secure the plate to the vertebral column.

In the second stage, the dorsal part of the plate was attached to the appropriate spinous processes with hemostats when the surgeon was satisfied with the position and security (Figure 6). Holes in the dorsal part of the plate were designed to allow for either 2.0 mm or 2.7 mm monocortical screws to be placed through them and into the spinous process with washers. Using these holes and either a 1.5 mm or 2.0 mm bit as appropriate, holes were then drilled in the spinous processes and monocortical screws placed with washers. Screws were placed individually without being fully tightened, with final tightening of all screws at the end of this stage. This secured the plate to the vertebral column in such a way as to allow some variation in individual screw placement while still providing a secure final attachment (Figure 7).

**Figure 8:**
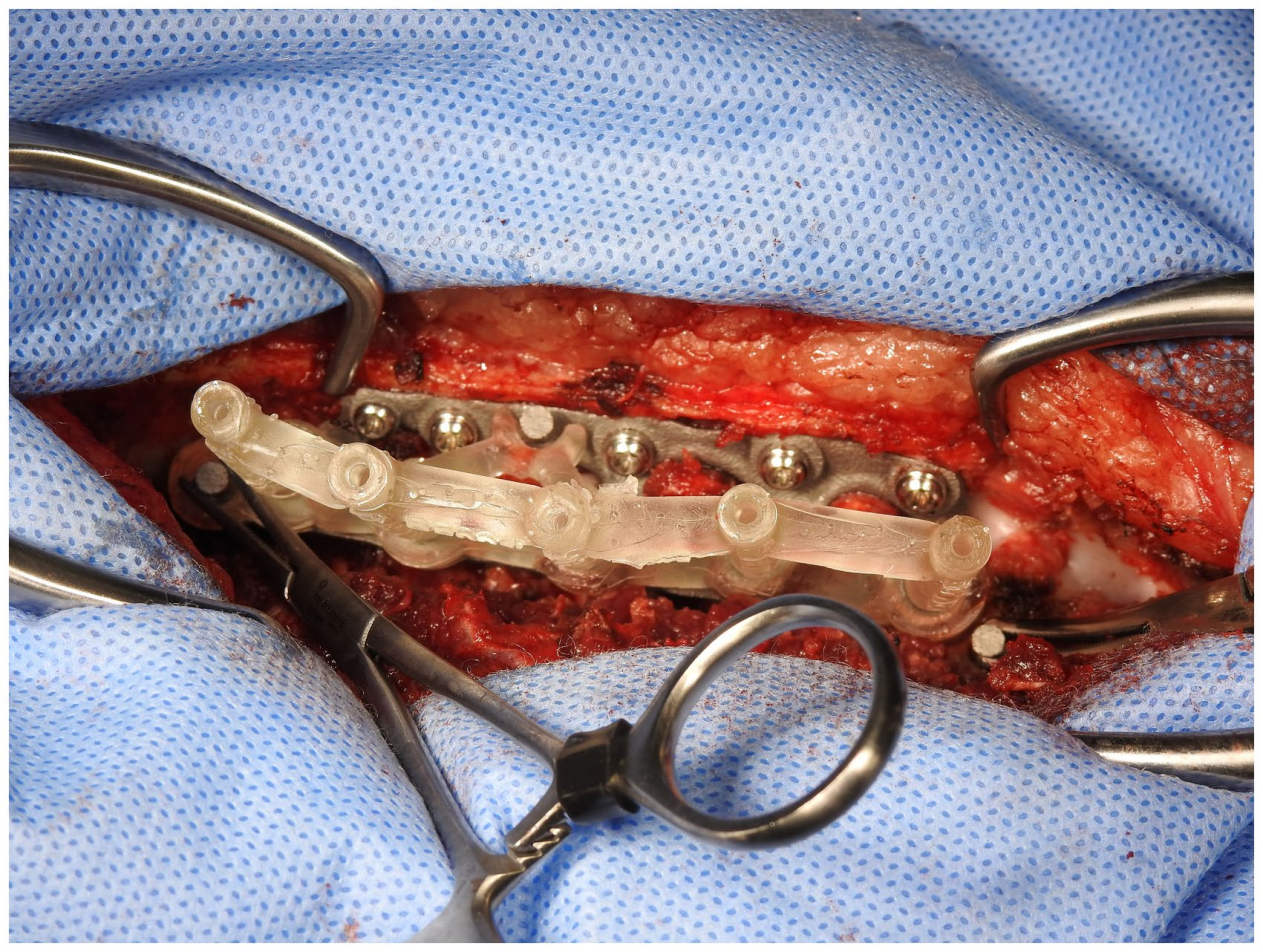
Curved haemostats clamped onto posts on spinal plate.

**Figure 9:**
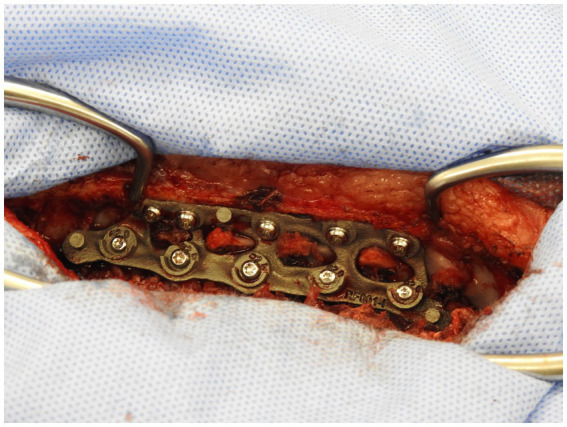
Plate screwed onto vertebral column prior to closure.

In the third stage, the stackable drilling guide was fitted to the plate. Specific posts created on the plate allowed for curved hemostats to be placed on the post over the drill guide to ensure a tight and secure fit while drilling was performed (Figure 8). During the plate design process, screw diameter, screw length and total drilling depth were measured, and these measurements allowed for accurate drilling of the screw holes into vertebral bodies. Once all screw holes were drilled, the drill guides were removed. Either 2.0 mm or 2.7 mm monocorical screws were then placed into the appropriate holes and tightened. Final tightening of all screws was then performed, and the surgery site was lavaged with warmed isotonic saline solution before routine closure (Figure 9).

### 2.5 Post-operative care and follow-up

**Figure 10:**
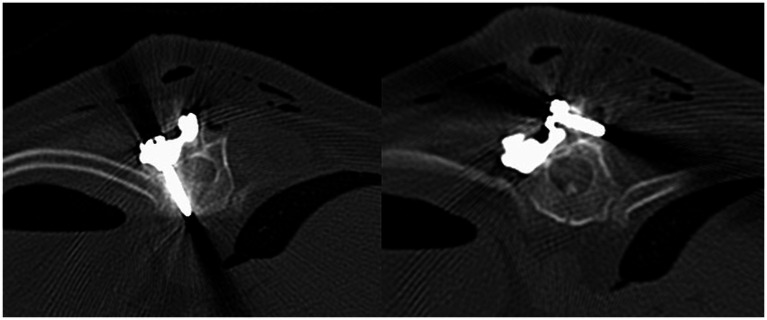
Transverse computed tomography images. Monocortical pedicle screw and spinal plate (left) and monocortical screw in spinous process and spinal plate (right).

Post-operative CT scan was performed after surgery to assess spinal plate and screw placement (Figure 10). Dogs were hospitalized overnight and administered Fentanyl CRI (3ug/kg/h). Clinical examination and neurological examination were conducted on the patients during their overnight hospitalization period and any declination or improvement was reported. Discharge was typically within 2 days and re-examination typically took place 2 weeks post-operatively. Gabapentin and meloxicam administered with discharge and a recommendation of restricted activity for 6 weeks post-operatively was typically made. A combination of physiotherapy and hydrotherapy was adopted for post-operative rehabilitation and recovery.

## 3 Results

### 3.1 Patients selected

Eleven dogs were included in this case series. Eight were brachycephalic. There were eight males (six castrated) and three females (all spayed). A median age of 8.5 years (range: 1 to 12 years) and a median bodyweight of 10.5 kg (range: 7 to 15.1 kg). Table 3 contains a comprehensive listing of the signalment, neurological signs (graded using a MFS classification scale), and duration of clinical signs for all dogs.

**Table 3:** Signalment, clinical signs and duration of clinical signs prior to initial neurological consultation.

Dog	Breed Bodyweight (kg) age (years), sex	Neurological signs	Clinical examination localisation	Duration of clinical signs
1	Japanese Spitz 7 kg, 12yo, MC	Progressive hindlimb paraparesis and proprioceptive hindlimb deficits.	T3-L3 myelopathy	2 years
2	Pug 7.2 kg, 9yo, FS	Non-progressive hindlimb paraparesis/ataxia and proprioceptive hindlimb deficits. Mild/moderate atrophy of caudal aspect of both hindlimbs. Fecal and urinary incontinence	T3-L3 myelopathy	8.5 years Acute onset
3	French bulldog 9.2 kg, 7yo, MC	Ambulatory with moderate progressive ataxia. Occasionally weight-shifts completely to thoracic limbs. Hyper-reflexic patellar reflexes. Cutaneous trunci cut-off around mid-lumbar on right, normal level on left.	T3-L3 myelopathy	1 month
4	Bull terrier 15 kg, 3yo, M	Progressive hindlimb paraparesis/ataxia and proprioceptive hindlimb deficits. Intermittent paraphimosis.	T3-L3 myelopathy	2 years
5	Jack Russell Terrier 9.5 kg, 1yo, FS	Progressive hindlimb paraparesis/ataxia and proprioceptive hindlimb deficits. Subtle head tilt.	T3-L3 myelopathy	9 months
6	French bulldog 11 kg, 10yo, MC	Progressive hindlimb paraparesis/ataxia and proprioceptive hindlimb deficits. Fecal incontinence	T3-L3 myelopathy	2 years 2 months
7	French bulldog 15.1 kg, 1yo, MC	Intermittent progressive lameness and ataxia in hindlimbs.	T3-L3 myelopathy	9 months
8	French bulldog 11.4 kg, 8.5yo, MC	T11-T13 and T7-T8 hemivertebrae diagnosis via radiographs. Progressive paraparesis. Fecal incontinence. Moderate hindlimb muscle atrophy. Pain on palpation of thoracolumbar junction.	T3-L3 myelopathy	2 years
9	Pug X Cavalier 10.8 kg, 6yo, FS	Progressive paraparesis and hindlimb ataxia. Fecal incontinence.	T3-L3 myelopathy	6 months
10	Pug 10.5 kg, 9yo, M	Progressive paraparesis, hindlimb ataxia and dropped tail. Fecal incontinence.	T3-L3 myelopathy	8 months
11	Pug 9.6 kg, 11yo, MC	Progressive hindlimb ataxia and hindlimb proprioceptive deficits. Fecal incontinence	T3-L3 myelopathy	6 weeks

### 3.2 Clinical signs

Ten dogs presented with a history of chronic progressive paraparesis with a median time of 0.8 years from onset of symptoms to initial neurological consultation (range: 1 month to 2 years). Two dogs presented with non-progressive paraparesis. Fecal incontinence was reported in six dogs and urinary incontinence in one dog. Nine dogs presented with proprioceptive deficits in their pelvic limbs. All dogs were diagnosed with a T3-L3 myelopathy based on neurological examination. Ten dogs were classified as MFS grade 2 prior to surgery; one dog was classified as grade 3. Table 3 shows the clinical signs, pre-operative neurological assessment, and neurolocalization for each patient that was noted in clinical examination.

### 3.3 Pre-operative diagnostic imaging findings

Table 4 shows pre-operative imaging findings for all dogs. Wedge vertebrae were found in three dogs and hemivertebrae were found in three dogs. Caudal articular process dysplasia (CAPD) was found in five out of eleven dogs and the most common site for CAPD was T12, followed by T11, T13 and T10. CAPD was the only vertebral malformation identified in five out of the eleven dogs. Other conditions identified by pre-operative CT and MRI include subarachnoid diverticulum and intervertebral disc protrusion. One dog had a subarachnoid diverticulum and five dogs had intervertebral disc protrusion. Two dogs had multiple intervertebral disc protrusions identified prior to surgery. T2W hyperintensity was identified in three out of eleven dogs. Where T2W hyperintensity was identified, it was typically adjacent to, or at the same site as, the CAPD or hemivertebrae lesions - likely due to the instability caused by these malformations.

**Table 4:** Neurolocalization, pre-operative imaging findings and neurological signs pre- and post-surgery.

Dog	MFS pre-operative / post-operative / follow up	CT/MRI findings, location of CVM, other findings	Neurological status prior to surgery	Surgery	Neurological status post-surgery	Neurological status at follow-up
1	2/2/2	MRI: T13 CAPD, L1 mild–moderate IVD protrusion/hypertrophy, L1 spinal cord gliosis or oedema, T2W hyperintensity. CT: T13 CAPD	Slight delay in advancement of both hindlimbs when walking. Mild wide-based stance. Mildly delayed proprioception in both hindlimbs.	T11-L1 plate stabilization.	No change from pre-op.	Scuffing LHL but moving better than prior to surgery
2	2/3/2	MRI: T13-L1 IVD protrusion/extrusion, T10-T11 T2W hyperintensity within spinal cord. CT: T10-T11 AP hypoplasia.	Bilateral hindlimb ataxia. Broad-based hindlimb stance. Delayed proprioception in both hindlimbs.	T9-T11 plate stabilization.	Neurological regression – non-ambulatory paraparesis. No voluntary urination (UMN bladder).	Ambulatory and weight bearing but hindlimb ataxia persists.
3	2/2/ (n/a)*	MRI: Kyphosis-lordosis with T12-T13 intervertebral disc protrusion (mild/moderate), L7-S1 intervertebral disc protrusion. CT: L5 wedge vertebrae, kyphosis caudal thoracic spine/vertebral malformations caudal thoracic spine Radiographs: L5 wedge vertebrae, kyphosis caudal thoracic spine/vertebral malformations caudal thoracic spine (butterfly/wedge/fused vertebra). Mild subluxation of left coxofemoral joint, mild left femoral neck remodeling.	Mild proprioceptive ataxia and proprioceptive deficits in both hindlimbs.	T11-L1 plate stabilization.	No change from pre-op.	Follow up information not available
4	2/2/ (n/a)	MRI: T2W hyperintensity at T8-T10. Radiographs/CT: T8-T10 vertebral anomaly, kyphosis	Bilateral severe hindlimb ataxia. Panniculus reflex absent from thoracolumbar junction on right.	T8-T11 plate stabilization.	No change from pre-operative condition.	Follow-up information not available.
5	3/3/3	Radiographs: T7 wedge vertebra, lumbosacral transitional vertebra, possible L4-L6 endplate lysis, T6-T7 kyphosis and intervertebral disc space narrow ventrally and wide dorsally. L7 sacralised. CT: Severe kyphosis/lordosis at T6-T7 intervertebral disc. Pre-mature closure of T7 vertebral endplate.	Non-ambulatory paraparesis and increased extensor tone and increased patella reflexes in both hindlimbs.	T5-T7 plate stabilization.	Non-ambulatory and less movement than pre-surgery.	Slight increase in tone and strength in both hindlimbs. Proprioception absent, reflexes normal to increased and motor function present at right hip. Stable post-surgery.
6	2/2/2	MRI/CT: IVD protrusion at T12-T13, T12-T13 CAPD. Hemi and wedge vertebrae at multiple sites along the spine.	Hindlimb ambulatory paraparesis and delayed proprioception in both hindlimbs. Hopping worse than paw placement.	T11-L1 plate stabilization.	Mild hindlimb proprioceptive deficits with mild hindlimb wide stance. Proprioception normal in left hindlimb but delayed in right hindlimb.	Right HL took longer to recover than left HL. Only one episode of dropping stools (incontinence).
7	2/2/ (n/a)	MRI: T7 hemivertebra, Kyphosis/lordosis over T7 ventral body, intraarachnoid diverticulum over left lateral surface of T8 spinal cord segment. CT: T7 hemivertebrae, kyphosis/lordosis over T7 ventral body.	Mild hindlimb paresis and delayed proprioception in both hindlimbs.	T6-T8 plate stabilization.	Mild hindlimb ataxia but walking well.	Follow up information not available.
8	2/2/2	MRI/CT: Left lateralized T12/T13 intervertebral disc extrusion with marked spinal cord compression. Caudal thoracic hemivertebrae and kyphosis/lordosis. L7/S1 intervertebral disc protrusion with foraminal stenosis bilaterally.	Ambulatory with moderate proprioceptive para-ataxia and paraparesis. Absent paw replacement and lateral hopping on the right pelvic limb. Delayed paw replacement and lateral hopping on the left pelvic limb.	T11-L1 plate stabilization.	Weakly ambulatory para-paretic and hindlimb ataxia.	Ambulatory with moderate to marked paraparesis and proprioceptive ataxia. Delayed to absent paw replacement in both hindlimbs. Moderate tone in both pelvic limbs.
9	2/2/2	MRI: T11-T12 and T12-T13 chronic intervertebral disc change. CT: T11-T12 and T12-T13 CAPD.	Mild–moderate decrease in proprioception and ataxia in both hindlimbs.	T11-T13 plate stabilization.	Weakly ambulatory and no gait improvement.	Neurological examination the same as pre-op.
10	2/2/1	MRI/CT: T11-T12 spinal cord compression secondary to CAPD.	Ambulatory with mild bilaterally symmetrical pelvic limb paresis. Pelvic limb reflexes were increased to normal. Mild thoracolumbar pain on palpation.	T10-T11 plate stabilization.	Fecal incontinence worsened but no change in hindlimb neurological function.	Good walking and movement, no signs of post-operative pain/neurological deficits.
11	2/2/2	MRI: T2W hyperintensity within spinal cord at T10-T11 IVD space level. T10 and T11 CAPD. CT: T10-T11, T11-T12 and T12-T13 CAPD.	Mild bilateral hindlimb ataxia and proprioceptive deficits. Normal to increased reflexes in both hindlimbs.	T9-T12 plate stabilization.	Paraparesis and hindlimb ataxia worsened.	Slightly improved hindlimb ataxia.

*(n/a) – no follow up MFS was available.

### 3.4 Surgical procedure

Surgery was performed as described. A left-sided dorsolateral approach was used in the majority of dogs. A midline dorsal approach was used for three dogs and a right-sided dorsolateral approach in dog 10. The plate was placed in the thoracolumbar region in all dogs, ranging from T5 cranially to L1 caudally. Table 4 shows the location in which the plate was placed for all patients.

If required, additional surgical procedures were undertaken by the surgeon (SL) and are outlined as follows: The articular facets of T12-T13 and T13-L1 in dog 3 were removed by high-speed burr. The T12-T13 articular facet process and both T12 and T13 pedicles were also removed in dog 8. Dog 8 had an acute Hansen type 1 IVD extrusion with moderate extradural hemorrhage at the level of T12/T13 which was incised with a sharp nerve hook and debulked but was not removed due to adhesions with the spinal cord. A right hemilaminectomy window was drilled into T8 in dog 7 and a cruciform incision made over the intra-arachnoid diverticulum, and a large amount of CSF was removed. The diverticulum was then marsupialized.

No significant intra-operative complication was observed in any of the surgeries performed however, dog 10 experienced intermittent episodes of bradycardia throughout the surgery and was administered atropine to increase heart rate. Additionally, the spinal plate was slightly difficult to place for dog 5, which is suspected to be due to the growth of the patient during the timeframe in which the surgery took place and the pre-operative measurements for plate creation occurred. All dogs had an uneventful recovery from surgery and anesthesia and CT scan was conducted for all dogs post-operatively to confirm appropriate plate and screw placement.

### 3.5 Post-operative imaging and spinal canal screw perforation

The post-operative CT scan for each patient was examined retrospectively by two of the authors (PG and SL) to assess the degree of screw breach. The degree of screw breach was classified using the modified Zdichavsky classification as outlined in Table 2 (36). A total of 38 screws were assessed across all patients included in this study. There was optimal screw placement (grade I) in 24/38 (63.2%) while partial penetration of the medial wall (grade IIa) was observed in 1/38 (2.6%) screws, partial penetration of the lateral wall (grade IIIa) was observed in 11/38 (28.9%) screws and full penetration of the lateral pedicle wall (grade IIIb) was observed in 2/38 (5.3%) screws. There were no screws observed in the CT scans to have full penetration of the medial vertebral wall (grade IIb).

### 3.6 Surgical outcomes

All dogs were ambulatory and had either maintained or improved neurological function at the time of the last post-operative neurological examination. However, there was neurological regression at discharge for five of the dogs whilst four dogs did not have any change in neurological function and two dogs had improved neurological function. Surgical outcomes immediately post-surgery and at follow-up examination are shown in Table 4.

Upper motor neuron bladder was observed in dog 2 post-operatively and manual bladder expression with subsequent placement of an indwelling urinary catheter was required. Additionally, dog 2 experienced aspiration pneumonia and a persistent azotemia which was subsequently diagnosed as chronic renal failure. As a result, dog 2 was hospitalized for 9 days post plate stabilization surgery which was the longest post-operative hospitalization period for any of the patients included in this study. All other dogs were discharged within 2 days of surgery.

## 4 Discussion

In this case series we describe the pre-operative findings, surgical protocol, and outcome of eleven dogs which underwent spinal stabilization using custom 3D-printed drill guides and titanium plates. Previous studies have demonstrated the safety and efficacy of 3D-printed drill guides for spinal stabilization in dogs to varying degrees. Elford et al. (46) reported 96.7% of optimal screw placement (grade I). Similarly, Violini et al. (36) reported 84% of screws drilled with optimal placement and 15% of screws to have penetrated the vertebral canal. In contrast, Guevara et al. (32) found that only 63.6% of pins were optimally placed and only 1% of pins violated the vertebral canal when placed with the assistance of a 3D-printed drill guide. Our results are similar to Guevara et al. (32) where we classified 63.2% of screws as having optimal placement whilst 2.6% of screws penetrated the medial vertebral wall and into the vertebral canal. A possible cause for this variation could be explained by differences in sample size and surgeon experience. As discussed by Violini et al. (36), the initial part of the surgery whereby the soft tissue is elevated from the cortices tends to result in significant variation between surgeons. This can subsequently impact the fit of the custom titanium plate and 3D-printed drill guide to the patient’s spine and therefore screw placement. In this study, one surgeon (SL) conducted all the stabilization surgeries and as such, less variation between surgical technique may have occurred, possibly contributing to the lower rates of spinal canal perforation. Median post-operative hospitalization length in this study was 2 days (range: 1 to 9 days) which was similar to the median 3-day hospitalization length reported by Violini et al. (36).

**Figure 11:**
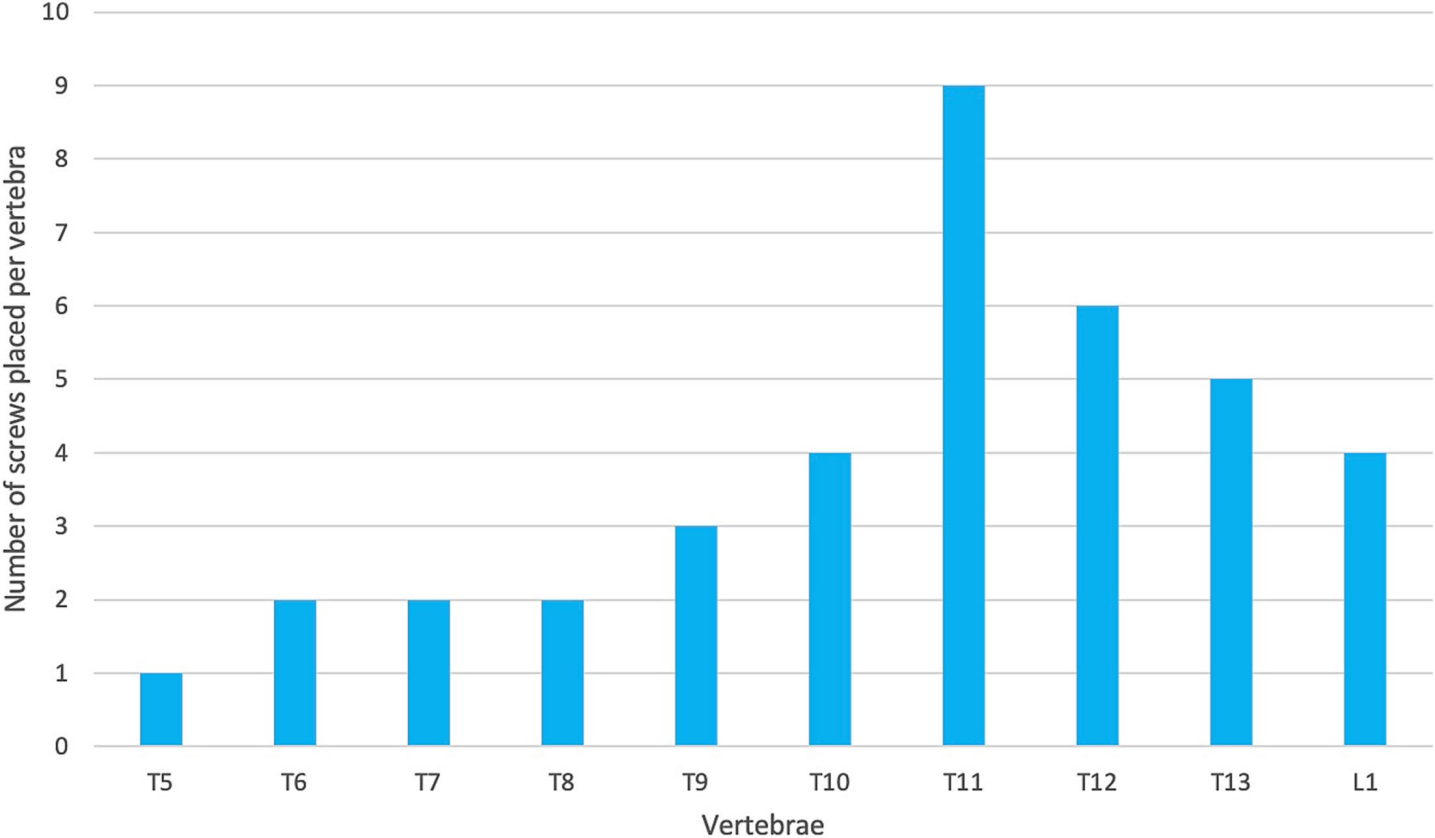
Frequency of screw placement in the vertebrae across all patients.

The accuracy of screw placement can also be impacted by surgical location within the vertebral column. Screw and pin placement within the caudal thoracic vertebrae is typically considered to be more challenging compared to implant placement in the cranial lumbar region (32). This is because screws drilled into these vertebrae need to be placed in the vertebral body through the vertebral pedicle when compared to the lumbar vertebrae where the screws can be placed directly into the vertebral body (37). Another such reason is that the thoracic vertebrae are closer to the chest and have narrower and steeper bone corridors (32, 38). Additionally, the presence of the anticlinal vertebra (T10-T12) can make dissection of soft tissues and drill guide placement challenging (32). In general, techniques involving 3D-printed drill guides rely on meticulous exposure and cleaning of soft tissues from bone surfaces to achieve good registration with the associated guides. Therefore, the quality of this dissection dictated to a large extent the accuracy of guide placement. In Elford et al. (46), the spinous processes of the more cranial thoracic vertebrae were removed in order to allow for appropriate drill guide placement and placement of bilateral screws. This was not undertaken in the present study as the design of the drill guide and plate meant that screws were only placed unilaterally. This variation in surgical methodology may have resulted in the variance of implant placement between the studies. Additionally, the most cranial thoracic vertebra stabilized by Guevara et al. (32) was T10 whilst the majority (42/60) screws placed by Elford et al. (46) were cranial to T10 (between T5-T9). In the present study, six of the eleven dogs had regions of implant placement from T10 caudally and nine out of eleven dogs had screws placed in either T10 or T11 (Figure 11). It is postulated that the variation in implant location may have contributed to the variation between the studies. This suggests that surgical location can impact optimal screw placement and could provide a possible explanation for a different rate of optimal screw placement.

Subjectively there were several advantages to the 3D-printed plates. The surgery was remarkably straightforward and subjectively patients recovered much more rapidly and with less postoperative discomfort than with other (more traditional) stabilization techniques. In addition, the placement of the dorsal screws into the spinous processes had a tendency to angle the abaxial surface of the plate dorsolaterally in such a way as to make subsequent drilling move away from the spinal cord if any placement error occurred. This increased the safety margin of the whole technique. An additional advantage of the custom titanium spinal plates is that they induce a lesser degree of beam-hardening artefact when compared to other materials such as stainless steel (39–41). This allows the patient to undergo post-operative CT scan for more accurate implant placement assessment. Additionally, MRI can also be safely conducted with titanium implants and will allow flexibility with available imaging modalities options should future imaging be required for patients (42). However, the design and production process for the plates (including time taken for quality assurance checks and delivery) on occasion took up to 4 weeks in duration, making this procedure a relatively poor option for the treatment of acute spinal cord conditions (eg traumatic spinal fracture). In the authors’ opinion this was the only drawback of this technique.

All methods of spinal stabilization, with the exception of spinal stapling, require screws or pins to be placed into the vertebrae of patients. As such, placing screws and/or pins into the vertebral bodies of patients is common practice during spinal stabilization. Two common plate systems used for canine spinal stabilization are String of Pearls (SOP; Orthomed UK Ltd., Halifax, West Yorkshire, UK) and Locking Compression Plates (LCP; DePuy Synthes, West Chester, PA). Both plate systems employ a fixed screw trajectory system that are perpendicular to the plate in order to allow for proper engagement with the locking mechanism. This poses challenges as plates are fixed in position as soon as the first screw is engaged and locked, making subsequent changes to screw trajectory difficult. As such, planning and exact screw placement are important in avoiding mispositioned locking plates due to inappropriately placed screws. In the present study, it is hypothesized that the use of custom 3D-printed plates allowed for better contact with the bony anatomy of the patient’s vertebrae and therefore ensured a more secure fit.

Another factor to consider when assessing surgical procedures is surgical time. When comparing time taken to place pins and screws with either freehand drilling or 3D-printed drill guides, a recent systematic review found that the use of drill guides allowed for more rapid placement of pins and therefore shortened surgical times in humans (43). This is important as previous studies have demonstrated that a negative association exists between total anesthesia duration and ambulation following spinal surgery, therefore impacting patient outcomes and prognosis (44). As such, differences between surgeon experience and approach can influence anesthesia duration and therefore patient outcomes reported. This was not a factor in the present study as one surgeon was responsible for all surgeries reported in this case series. However, future studies should investigate ideal surgical duration for optimum surgical outcomes for patients undergoing this procedure.

The modified Zdichavsky classification system was selected to grade screw placement in this study due to its high intra- and inter-observer reliability reported previously as well as its use in comparable studies (24, 36, 45, 46). To the knowledge of the authors, there is currently no standardized screw placement classification system that exists and accepted for veterinary patients (45–47).

A theoretical disadvantage of spinal stabilization, particularly in cases of multi-vertebral stabilization, is adjacent segment disease (48, 49). Dynamic stresses are redirected to adjacent intervertebral discs which can cause or accelerate the development of intervertebral disc disease. This process is also known as the domino effect and may impact the long-term efficacy of the surgery. Future research should investigate long term effects of this surgical technique and whether adjacent segments to the location of spinal stabilization are at an increased risk of intervertebral disc disease development. Although, since many brachycephalic breeds are genetically predisposed to intervertebral disc disease, it may be difficult to differentiate between disease development due to adjacent segment disease and disease development that would otherwise have occurred without spinal stabilization.

The limitations encountered during this study can be used to improve the design for future studies. The limitations of this study include its retrospective nature and the small number of dogs that were included. A prospective, randomized case–control study based on a larger sample size of dogs diagnosed with spinal instability due to congenital vertebral malformations would be required to overcome these limitations. Another limitation is the lack of longer-term outcomes for many of the dogs included in this study. It would be interesting to see the long-term efficacy of the surgical procedure and whether any vertebral instability and neurological regression would be likely to recur.

## 5 Conclusion

The use of custom 3D-printed drill guides and titanium spinal plates can be a safe and more straightforward surgical spinal stabilization alternative for patients with thoracolumbar malformations leading to spinal instability. All dogs in this study either maintained or improved neurological function approximately two weeks post-operatively. Although further investigation into long-term prognosis and consequences for this surgical technique is required, the authors found that ease of application, reduction in post-operative hospitalization and likelihood of short-term stabilization or improvement of neurological signs to be promising.

## Data Availability

The original contributions presented in the study are included in the article/supplementary material, further inquiries can be directed to the corresponding author/s.
